# Antibacterial, Antifungal, and Antiviral Bioactive Compounds from Natural Products

**DOI:** 10.3390/molecules29040825

**Published:** 2024-02-11

**Authors:** Xun Song

**Affiliations:** College of Pharmacy, Shenzhen Technology University, Shenzhen 518118, China; xsong@szu.edu.cn

## 1. Introduction

In the relentless pursuit of innovative therapeutic agents, natural products have emerged as a transformative avenue in the battle against infectious diseases. This Special Issue, entitled “Antibacterial, Antifungal, and Antiviral Bioactive Compounds from Natural Products”, seeks to unravel the potential within nature’s pharmacopeia and its ability to provide a rich source of diverse compounds with remarkable antimicrobial properties [1]. Historically integral to traditional medicine, natural products offer a palette of compounds, including alkaloids, flavonoids, terpenoids, and peptides from various sources, showcasing the versatility of nature’s defenses against bacteria, fungi, and viruses [2].

The increasing threat of drug-resistant microorganisms necessitates innovative approaches, and natural compounds present a promising solution [3]. Studies have highlighted their efficacy against a spectrum of bacteria, addressing the multifaceted nature of bacterial infections. Beyond conventional antifungal agents, fungal infections prompt the exploration of natural products from fungi, plants, and bacteria that exhibit a range of antifungal activities. This diversity broadens the scope for discovering new antifungal drugs to combat emerging threats.

In the realm of antiviral research, natural products demonstrate inhibitory effects against various viruses; this is vital in addressing the evolving landscape of viral infections, which is exemplified by global pandemics [4]. Derived compounds offer a foundation for understanding viral interactions and developing targeted interventions.

As we embark on this exploration of bioactive compounds, our aim is to unveil not only their therapeutic potential, but also the intricate mechanisms through which nature defends against microbial adversaries. Additionally, this Special Issue discusses a range of antimicrobial natural products with other biological functions, thus further highlighting the diverse capabilities within nature’s pharmacopeia.

## 2. An Overview of Published Articles

Xiaopeng Hu’s article [Contribution 1] discusses the potential inhibitors of the monkeypox virus. Monkeypox outbreaks pose a global health threat, exacerbated by the absence of effective medicines against orthopoxviruses. Molecular modeling, focusing on natural products like traditional Chinese medicine (TCM), has revealed potential inhibitors. Three compounds—rosmarinic acid, myricitrin, quercitrin, and ofloxacin—have demonstrated significant binding to monkeypox DNA topoisomerase I, suggesting promising antiviral effects. Molecular dynamics simulations support their stability. The study by Hu et al. highlights the potential application of these compounds as poxvirus inhibitors, emphasizing the need for further research to assess their therapeutic efficacy.

The review by Mariana Rodríguez-Cisneros et al. [Contribution 2] is focused on the antimicrobial compounds produced by *Burkholderia* Sensu Stricto. The rise in multi-drug-resistant microorganisms is currently demanding the exploration of novel antimicrobial compounds. The World Health Organization has highlighted that *Acinetobacter baumannii*, *Pseudomonas aeruginosa*, and *Enterobacteriaceae* are critical bacteria that require urgent eradication. *Burkholderia*, known for its production of antimicrobials, offers a diverse array of compounds, including N-containing heterocycles, polyenes, bacteriocins, and more. These compounds exhibit potential not only as antimicrobials against bacteria and fungi, but also as candidates for anticancer or antitumor agents. The review comprehensively explores Burkholderia’s extensive repertoire of antimicrobial compounds, with a focus on those tested in vitro, and includes information on novel compounds discovered through genome-guided approaches.

The review by Meijie Xu et al. [Contribution 3] highlights the 185 antimicrobial natural products that had been extracted from *Fusarium* strains by 2022, showcasing their antibacterial, antifungal, antiviral, and antiparasitic effects. The comprehensive analysis emphasizes the importance of *Fusarium* as a valuable reservoir for new bioactive secondary metabolites. The review concludes by proposing methodologies for the efficient discovery of natural products obtained from *Fusarium* strains.

The fourth article published in this Special Issue is by Ting-Ting Tang et al. [Contribution 4] and focuses on the use of flavonoids obtained from *Scutellaria barbata* D. Don (SB) as inhibitors of HIV-1 and cathepsin L proteases. SB is a medicinal plant rich in flavonoids, and is known for its antitumor, anti-inflammatory, and antiviral properties. The study evaluated SB extracts and identified nine flavonoids that inhibit HIV-1 and SARS-CoV-2 proteases. The results revealed that scutellarein is a lead compound with potent dual inhibitory activity against both proteases. The study emphasized the importance of specific hydroxyl group introductions in flavones regarding the enhancement of anti-protease activities. Notably, luteolin exhibited the potent and selective inhibition of HIV-1 protease. These findings suggest the potential use of SB-derived flavonoids as promising candidates in the development of effective dual protease inhibitors.

In their study, Ben Chung-Lap Chan et al. [Contribution 5] create a vaccine-like supplement against respiratory infection using recombinant *bacillus subtilis* spores expressing SARS-CoV-2 spike protein with natural products. This study explores an alternative approach to COVID-19 vaccination using engineered *Bacillus subtilis* to produce “S spores” that mimic the SARS-CoV-2 spike protein. Administered orally with the natural adjuvants *Astragalus membranaceus* and *Coriolus versicolor*, the S spores induced mild immune responses against COVID-19 without toxicity. Co-administration enhanced the mucosal IgA responses, while prior oral inoculation expedited and strengthened the IgG responses when followed by the commercial vaccine CoronaVac. In vitro studies demonstrated immune activation by AM, CV, and *B. subtilis* spores. This novel combination shows potential in the development of a supplementary vaccine against respiratory infections, addressing concerns regarding needle aversion and side effects.

In the sixth article, Ming-Feng He [Contribution 6] focuses on the antiviral mechanism of the alkaloid coptisine against influenza virus. This study identifies that coptisine, a compound found in Chinese herbs, is a potent inhibitor of the influenza virus, exhibiting an EC_50_ of 10.7 μM in MDCK cells. Coptisine’s antiviral effects are attributed to its upregulation of the p21 signaling pathway, leading to the increased expression of p21 and FOXO1. The compound’s effectiveness is highlighted in pre-treatment, showing a superior reduction in viral replication compared to co-treatment or post-treatment. Docking analysis suggests that coptisine inhibits MELK activity, forming hydrogen bonds in the catalytic pocket. These findings position coptisine as a promising antiviral agent with the ability to regulate the p21 pathway to combat viral infections.

The research presented by Guangxin Chen [Contribution 7] delves into the antioxidant and anti-inflammatory effects of cepharanthine (CEP). CEP, a biscoclaurine alkaloid extracted from *Stephania cepharantha* Hayata, has been widely utilizes in the treatment of various acute and chronic diseases, including leukopenia, and snake bites. The study explores the therapeutic potential of CEP in countering oxidative stress and inflammation. CEP effectively alleviates weight loss, reduces disease activity, and maintains intestinal integrity in colitis mice. It mitigates malondialdehyde levels, boosts the glutathione content, and inhibits inflammatory responses. Additionally, CEP activates the AMPK-α1/AKT/GSK-3β/NRF2 signaling pathway while suppressing MAPKs and NF-κB p65 pathways. The protective effects extend to leukopenia and snake bites but are compromised in NRF2 knockout mice. This research underscores that CEP is a promising therapeutic agent against oxidative stress and inflammation, emphasizing the potential use of NRF2 as a target for treating inflammatory bowel disease.

Lihan Zhao’s study [Contribution 8] focuses on the isolation and anticancer progression of the chemical constituents of *Bridelia balansae* Tutcher. The study highlights the anticancer potential of the dichloromethane extract obtained from *Bridelia balansae* Tutcher roots against HCT116 colorectal cancer cells. Fourteen compounds were identified, including a novel aryltetralin lignan; this was 4′-demethyl-4-deoxypodophyllotoxin (**1**), whose stereochemistry was validated via X-ray crystallography. Compound **1** exhibited significant cytotoxicity (IC_50_ at 20 nM) through the induction of apoptosis, reducing HCT116 cell migration and downregulating MMP2 and p-Akt, and upregulating p21. This comprehensive analysis positions *B. balansae* as a valuable source of potential anticancer lead compounds, emphasizing the therapeutic promise of compound **1** against the proliferation and metastasis of cancer.

Ruirui Yu’s work [Contribution 9] focuses on the isolation and identification of the chemical compounds obtained from *Agaricus blazei* Murrill and their in vitro antifungal activities. This research delves into the antifungal properties of *A. blazei*, an esteemed medicinal and edible fungus. Six isolated compounds from *A. blazei*, including linoleic acid, 1,1′-oxybis(2,4-di-tert-butylbenzene), glycerol monolinoleate, volemolide (17R)-17-methylincisterol, (24s)-ergosta-7-en-3-ol, and dibutyl phthalate, were identified and evaluated for their antifungal activities against *Trichophyton mentagrophology*, *Trichophyton rubrum*, *Candida albicans*, and *Cryptococcus neoformans*. Notably, compound **2** displayed significant inhibition against *T. mentagrophology*, compound **3** against *T. rubrum*, and compound **6** against *C. albicans.* The findings underscore *A. blazei*’s medicinal potential as an antifungal agent, revealing promising avenues for further research.

Jinrong Lin et al. [Contribution 10] performed an evaluation of the anti-cancer activity of lasiokaurin (LAS), a natural antimicrobial diterpenoid, in a triple-negative breast cancer (TNBC) model. TNBC poses challenges due to its aggressiveness and limited treatment options. This study explores LAS as a promising anti-TNBC agent. LAS exhibits significant efficacy in inhibiting TNBC cell growth, with its induction of cell cycle arrest, apoptosis, and DNA damage, and suppression of metastasis. It targets multiple pathways, including PI3K/Akt/mTOR and STAT3, suggesting its potential application in multitarget therapy. Moreover, the application of LAS has been found toe safely reduce tumor growth in a mouse model, suggesting its utilization as a potent candidate for TNBC treatment.

## 3. Conclusions

In the face of infectious diseases, the urgent need for innovative antimicrobial agents is underscored by antibiotic resistance, persistent fungal infections, and the constant threat of viral outbreaks. The exploration of natural products as reservoirs of antibacterial, antifungal, and antiviral bioactive compounds has garnered significant attention, thus paving the way for potential breakthroughs in the combat of microbial pathogens.

### 3.1. Antibacterial Bioactive Compounds: A Nature-Inspired Arsenal against Bacterial Infections

Natural products appear to possess a diverse array of antibacterial compounds with therapeutic promise. Compounds such as alkaloids, flavonoids, and terpenoids derived from plants exhibit potent antibacterial activity against a spectrum of strains. Essential oils, exemplified by constituents like thymol and carvacrol, showcase remarkable efficacy against both Gram-positive and Gram-negative bacteria. Additionally, marine organisms have been found contribute unique antibacterial peptides, such as bacteriocins, that exhibit selective toxicity towards bacterial pathogens [5,6].

### 3.2. Antifungal Bioactive Compounds: Nature’s Defense against Fungal Pathogens

Fungal infections pose a persistent threat to global health, particularly in immunocompromised individuals. Natural products offer diverse antifungal agents that target various stages of the fungal life cycle. Polyphenols such as resveratrol and catechins disrupt fungal cell membranes, while secondary metabolites such as mycotoxins exhibit potent antifungal properties [7,8].

### 3.3. Antiviral Bioactive Compounds: Harnessing Nature’s Defense Mechanisms

The ongoing battle against viral infections requires the implementation of innovative antiviral strategies. The natural products that are derived from plants, including polyphenols, flavonoids, and alkaloids, interfere with viral processes such as entry, replication, and maturation. Marine organisms also contribute unique antiviral compounds that exhibit promise regarding the inhibition of viral infections, such as sulfated polysaccharides and lectins [9,10].

### 3.4. Mechanisms of Action: Unveiling the Versatility of Natural Bioactive Compounds

The success of natural bioactive compounds against microbial pathogens lies in their diverse mechanisms of action. Antibacterial compounds disrupt cell membranes, inhibit protein synthesis, or interfere with metabolic pathways. Antifungal agents target cell membranes, inhibit ergosterol synthesis, and affect specific cellular processes. Antiviral compounds interfere with the replication, assembly, or entry of the virus into host cells, providing a multi-pronged defense against viral infections.

### 3.5. Challenges and Future Directions: Toward Sustainable Antimicrobial Solutions

While the potential application of natural bioactive compounds is vast, addressing challenges such as bioavailability, standardization, and sustainability is crucial for their successful clinical translation. The development of resistance to natural compounds in microbial pathogens necessitates concerted efforts in research and development.

In conclusion, the exploration of the antibacterial, antifungal, and antiviral bioactive compounds obtained from natural products holds great promise for novel antimicrobial agents. Nature’s diverse arsenal, honed over millions of years of evolution, forms a foundation for innovative drug discovery. Their multifaceted mechanisms of action, coupled with recent advancements in extraction techniques and analytical methods, position natural products as invaluable elements in the battle against microbial infections. Interdisciplinary collaborations between chemists, biologists, and clinicians will play a pivotal role in fully unlocking the potential of these natural bioactive compounds, and pave the way for a sustainable and effective approach to global infectious disease management.
